# Phase I and pharmacological study of the new topoisomerase I inhibitor GI147211, using a daily x 5 intravenous administration.

**DOI:** 10.1038/bjc.1996.130

**Published:** 1996-03

**Authors:** C. J. Gerrits, G. J. Creemers, J. H. Schellens, P. Wissel, A. S. Planting, R. Kunka, K. Selinger, M. de Boer-Dennert, Y. Marijnen, M. Harteveld, J. Verweij

**Affiliations:** Department of Medical Oncology, Rotterdam Cancer Institute, The Netherlands.

## Abstract

Topoisomerase I inhibitors are interesting anti-cancer agents with a novel mechanism of action. We performed a phase I study with intravenous GI147211, a new semisynthetic camptothecin analogue, using a daily x 5 schedule administered every 3 weeks, to evaluate the side-effects and pharmacokinetics of the agent. Patients with a histologically confirmed diagnosis of a solid tumour refractory to standard froms of therapy were eligible for the study. GI147211 was given as a 30 min intravenous infusion daily for 5 consecutive days, repeated every 3 weeks. In subsequent patient cohorts the dose was escalated from 0.3 to 1.5 mg m-2 day-1. Pharmacokinetics analysis was performed on days 1 and 4 of the first course using a validated high-performance liquid chromatographic assay and non-compartmental methods. A total of 19 patients were entered into the study, one patient was not evaluable for toxicity because only one drug administration was given. Eighteen patients received a total of 67 courses through four dose levels. The dose-limiting toxicities were neutropenia and thrombocytopenia at the dose of 1.5 mg m-2 day-1. Nadirs occurred on day 15 and day 15 respectively. Other toxicities were mild and infrequent and included nausea/vomiting, headache and alopecia. The maximal tolerated dose was 1.2 mg m-2 day-1. One partial response was observed in a patient with colorectal cancer. The total plasma clearance was 999+/-184 ml min-1 (range 640-1329). The volume of distribution was 190+/-461 m-2 and the terminal half-life was 3.7+/-1.2 h. The AUC increased linearly with the administered dose. A steep and significant sigmoid relationship was established between the AUC and the percent decrease of ANC. GI147211 is a new topoisomerase I inhibitor that induced dose-limiting neutropenia and thrombocytopenia in this phase I study. The recommended dose for phase II studies with this schedule is 1.2 mg m-2 x 5 every 3 weeks.


British Journal of Cancer (1996) 73, 744-750
© 1996 Stockton Press All rights reserved 0007-0920/96 $12.00

### Phase I and pharmacological study of the new topoisomerase I inhibitor GI147211, using a daily ×5 intravenous administration

CJH Gerrits[1], GJ Creemers[1], JHM Schellens[1], P Wissel[2], AS Th Planting[1], R Kunka[2], K Selinger[2], M de Boer-Dennert[1], Y Marijnen[3], M Harteveld[1] and J Verweij[1]

[1]*Department of Medical Oncology, Rotterdam Cancer Institute, Rotterdam, The Netherlands;* [2]*Glaxo Wellcome Inc, Department of Clinical Pharmacology, NC, USA;* [3]*Glaxo Wellcome Inc, Department of Clinical Research, Zeist, The Netherlands.*

**Summary** Topoisomerase I inhibitors are interesting anti-cancer agents with a novel mechanism of action. We performed a phase I study with intravenous GI147211, a new semisynthetic camptothecin analogue, using a daily × 5 schedule administered every 3 weeks, to evaluate the side-effects and pharmacokinetics of the agent. Patients with a histologically confirmed diagnosis of a solid tumour refractory to standard forms of therapy were eligible for the study. GI147211 was given as a 30 min intravenous infusion daily for 5 consecutive days, repeated every 3 weeks. In subsequent patient cohorts the dose was escalated from 0.3 to 1.5 mg $m^{-2}$ $day^{-1}$. Pharmacokinetics analysis was performed on days 1 and 4 of the first course using a validated high-performance liquid chromatographic assay and non-compartmental methods. A total of 19 patients were entered into the study, one patient was not evaluable for toxicity because only one drug administration was given. Eighteen patients received a total of 67 courses through four dose levels. The dose-limiting toxicities were neutropenia and thrombocytopenia at the dose of 1.5 mg $m^{-2}$ $day^{-1}$. Nadirs occurred on day 15 and day 15 respectively. Other toxicities were mild and infrequent and included nausea/vomiting, headache and alopecia. The maximal tolerated dose was 1.2 mg $m^{-2}$ $day^{-1}$. One partial response was observed in a patient with colorectal cancer. The total plasma clearance was $999 \pm 184$ ml $min^{-1}$ (range 640–1329). The volume of distribution was $190 \pm 46$ l $m^{-2}$ and the terminal half-life was $3.7 \pm 1.2$ h. The AUC increased linearly with the administered dose. A steep and significant sigmoid relationship was established between the AUC and the per cent decrease of ANC. GI147211 is a new topoisomerase I inhibitor that induced dose-limiting neutropenia and thrombocytopenia in this phase I study. The recommended dose for phase II studies with this schedule is 1.2 mg $m^{-2}$ × 5 every 3 weeks.

**Keywords:** topoisomerase I; phase I study; GI147211

GI147211, [7-(methylpiperazinomethylene)-10,11-ethylenedioxy-20(S)] camptothecin dihydrochloride, is a water-soluble semisynthetic analogue of camptothecin (CPT). Early clinical trials with CPT in the late 1960s showed hints of activity of this plant alkaloid in a variety of solid tumours (Gottlieb *et al.*, 1970; Muggia *et al.*, 1972; Creaven *et al.*, 1972). Its further development was stopped because of unpredictable and severe myelosuppression, gastrointestinal toxicity and haemorrhagic cystitis.

In the late 1980s two discoveries brought about a renewal of interest in CPT; firstly, topoisomerase I was identified as the single cellular target of CPT (Hsiang *et al.*, 1985; Hsiang and Liu, 1988) and, secondly, an overexpression of topoisomerase I was found in various tumour cell lines but not in normal tissues (Giovanella *et al.*, 1989; Hirabayaski *et al.*, 1992; Potmesil *et al.*, 1988). Topoisomerase I is a nuclear enzyme that resolves topological problems of the torsionally strained (supercoiled) DNA (Slichenmyer *et al.*, 1993). This is achieved by forming a covalent adduct between topoisomerase I and the DNA, termed the cleavable complex. This catalytic intermediate creates single-strand DNA breaks, allowing the DNA molecule to rotate around the intact DNA strand at the cleavage site, leading to a relaxation of the DNA molecule and in this way replication, transcription and other DNA functions can proceed. These enzyme-bridged breaks are then resealed by topoisomerase I. CPT stabilises the cleavable complexes, thereby preventing resealing of single-strand DNA breaks in the presence of the drug (Covey *et al.*, 1989; D'Arpa and Liu, 1989; Eng *et al.*, 1988). Cytotoxicity is specific to the S-phase of the cell cycle because the double-strand breaks that occur during this phase are more difficult to repair in the presence of the drug (Horwitz and Horwitz, 1973).

Recently, several semisynthetic CPT analogues (Creemers *et al.*, 1994; Potmesil, 1994) have been developed, aiming at reduced toxicity and sustained or improved activity.

One of these analogues, GI147211, demonstrated significant cytotoxicity against several xenografts of human cancers including HT-29 and SW-48 colon, PC-3 prostate, MX-1 breast, H460 lung, SKOV3 ovarian and KB epidermoid carcinomas (Emerson *et al.*, 1993, 1994, 1995). The relative effect on tumour growth was dose-schedule dependent with a greater reduction in tumour volume achieved by prolonged dosing. $LD_{10}$ in mice was 75 mg $m^{-2}$ (20 mg $kg^{-1}$) given as a single bolus injection. Animal toxicology studies by the intravenous route showed that myelosuppression was the main toxicity and was dose-limiting.

We performed a phase I and pharmacological study with intravenous GI147211 on a daily × 5 regimen, repeated every 3 weeks, in patients with solid tumours.

### Patients and methods

### *Patient selection*

Patients with a histologically confirmed diagnosis of a solid tumour refractory to standard forms of therapy were eligible for this study. Other eligibility criteria included: (1) age ⩾ 18 years; (2) an Eastern Cooperative Oncology Group (ECOG) performance status ⩽ 2; (3) a predicted life expectancy of at least 3 months; (4) no previous anti-cancer therapy for at least 3 weeks (6 weeks for previous nitrosoureas or mitomycin C); (5) adequate haematopoietic (WBC ⩾ $3 \times 10^9$ $l^{-1}$, ANC ⩾ $1.5 \times 10^9$ $l^{-1}$ and platelets $100 \times 10^9$ $l^{-1}$), hepatic (bilirubin within normal limits, AST, ALT and/or alkaline phosphatase ⩽ 2.0 × normal), and renal (serum creatinine ⩽ 130 μmol $l^{-1}$) functions. All patients gave written informed consent.

Correspondence: CJH Gerrits, Department of Medical Oncology, Rotterdam Cancer Institute, Groene Hilledijk 301, 3075 EA Rotterdam, The Netherlands
Received 11 September 1995; accepted without revision; accepted 30 October 1995

### Treatment and dose escalation

In the published phase I studies (Rowinsky et al., 1992; Verweij et al., 1993) using topotecan on a daily × 5 schedule, the starting dose was 1/30th of the murine $LD_{10}$ level because of the significant interspecies differences in toxicity. By rough estimate the murine $LD_{10}$ for GI147211 was equivalent to topotecan. However, the *in vitro* and *in vivo* pharmacology suggested that topotecan is 2.2-fold less potent than GI147211 (15–16). Therefore it was felt that a safe starting dose for GI147211 should be less than 1/30 of the mouse $LD_{10}$; 0.3 mg $m^{-2}$ $day^{-1}$ given as a 30 min infusion for 5 consecutive days was selected. Courses were to be repeated every 3 weeks as tolerated. Dose escalations were based on the prior dose level toxicity. For example if no toxicity was seen in the prior dose, ⩽100% dose escalation was allowed. However, if toxicity was seen, a dose escalation of 25–50%, which was determined by the worst significant toxicity, was prescribed. At least three patients were entered at each dose level. The maximum tolerated dose (MTD) was defined as one dose level below the dose that induced dose-limiting toxicities (DLTs), which were defined as at least one of the following: (1) ANC$\leqslant 0.5\times10^9$ $l^{-1}$ or platelets$\leqslant 50\times10^9$ $l^{-1}$ for more than 5 days; (2) ANC$\leqslant 0.5\times10^9$ $l^{-1}$ with fever requiring parenteral antibiotics, and/or non-hematological toxicity⩾CTC grade 3 in more than one-third of GI147211 naive patients (at least two of a maximum of six patients). Intrapatient dose escalation was not performed.

GI147211 was supplied by Glaxo as a clear solution in vials of 2.0 ml. The vials contained a mixture of 0.5 mg of GI147211 and 100 mg of dextrose. The pH was adjusted to 3.5 with sodium hydroxide or hydrochloric acid. GI147211 was diluted in D5W. The infusion bag (GI147211 + D5W) contained 100 ml exactly.

During the first course patients were hospitalised, all other courses were given at the outpatient clinic.

### Treatment assessment

Before therapy medical history was taken and complete physical examination, complete blood cell (CBC) count, serum chemistries including sodium, potassium, chloride, bicarbonate, calcium, phosphorus, creatinine, urea, uric acid, glucose, total protein, albumin, bilirubin, alkaline phosphatase, AST, ALT, were performed, as were urinalysis, coagulation parameters (APTT, PT), electrocardiogram (ECG) and chest radiograph. Weekly evaluations between the courses included history, physical examination, toxicity assessment according to the CTC criteria (National Cancer Institute, 1988) and serum chemistries. CBC and urinalysis were determined twice weekly. Tumour measurements were performed after every two courses and evaluated according to the WHO criteria for response (World Health Organization, 1979); patients were taken off protocol in case of disease progression.

### Pharmacokinetics

For pharmacokinetic analysis whole blood samples (7 ml) in heparinised tubes were collected from an indwelling i.v. canula, placed in the arm contralateral to that receiving the drug, before infusion and at 15, 25, 45 min and 1, 1.5, 2, 4, 6, 8, 10, 12 h after the initiation of the infusion on days 1 and 4 of the first course. Urine was collected within a 2 hour interval before the dosing and over the intervals; 0–4, 4–8, 8–12 and 12–24 h. Both blood and urine samples were analysed for the lactone form using a validated chromatographic assay, according to a method published by Stafford and St. Claire (1995). The AUC was calculated using the trapezoidal method with extrapolation of the curve to infinity on day 1 and extrapolated to 24 h on day 4. The terminal half-life was calculated as $\ln 2/\lambda$ where $\lambda$ is the elimination rate constant, the total plasma clearance (Cl) as dose/AUC and the apparent volume of distribution at steady state ($V_{dss}$) as

$$\frac{\text{Dose}\times\text{AUMC}}{\text{AUC}^2}-\frac{\text{Dose}(0.5)}{2\text{AUC}}$$

Sigma Plot for Windows (release 2.0, Jandel scientific) and PCNONLIN (release 4.0, SCI software) were used for pharmacological data analysis. The sigmoid $E_{max}$ model (Hill equation) was used to explore relationships between pharmacokinetic parameters and per cent decrease ANC and per cent decrease thrombocytes. The Gauss–Newton algorithm was used without weighing factor. The concentration of the drug in the infusion bags was also quantitated by high-performance liquid chromatography (HPLC).

### Results

A total of 19 patients entered the study. Patient characteristics are given in Table I. All patients were eligible but one patient with NSCLC was considered not evaluable for toxicity and response as the patient was taken off study after the first drug administration, because of development of broncho-oesophageal fistula. In total 18 patients were evaluable for toxicity and response. The total number of evaluable courses was 67. The median number of courses per patient was four (range 4–10). Dose levels studied were 0.3, 0.6, 1.2 and 1.5 mg $m^{-2}$ day $^{-1}$.

### Hematological toxicity

Neutropenia and thrombocytopenia were the dose-limiting toxicities of GI147211 on this schedule (Table II). No myelotoxicity was observed at the first two dose levels. At the third dose level (1.2 mg $m^{-2}$ day $^{-1}$), grade 3–4 neutropenia and thrombocytopenia were seen in 7/15 and 6/15 of the courses respectively. The median ANC nadir at this dose level was $1.29\times10^9$ $l^{-1}$ (range 0.08–5.6), for platelets it was $74\times10^9$ $l^{-1}$ (range 33–379). The median duration of severe myelosuppression, expressed as the number of days between the first occurrence of grade 3–4 toxicity and recovery to⩽grade 2 toxicity, was 10 days for neutropenia and 7 days for thrombocytopenia (Table III). In three of six patients first receiving 1.2 mg $m^{-2}$ day $^{-1}$, the dose was subsequently reduced to 0.9 mg $m^{-2}$ day $^{-1}$; in two of the patients because of slow recovery from myelosuppression (with retreatment being permitted on day 28 and day 35 respectively); and in one patient because of febrile neutropenia with sepsis during the first course. At 1.5 mg $m^{-2}$ day $^{-1}$ the dose-limiting toxicity was reached (Figure 1). Grade 3–4 neutropenia was noted in 13/15 courses (86%). The median ANC nadir was $0.09\times10^9$ $l^{-1}$

**Table I** Patient characterisitics

| | |
|---|---|
| Number of patients | 19 |
| Sex (male/female) | 10/9 |
| Median age (range) | 59 (34–74) |
| Median performance score (ECOG) | |
| 0 | 11 |
| 1 | 8 |
| 2 | 0 |
| Prior therapy | |
| Chemotherapy | 6 |
| Radiotherapy | 1 |
| Both | 7 |
| None | 5 |
| Tumour types | |
| NSCLC | 2 |
| Colorectal cancer | 9 |
| Sarcoma | 3 |
| Unknown primary | 1 |
| Pancreatic cancer | 1 |
| Breast cancer | 1 |
| Mesothelioma | 1 |
| Oropharyngeal cancer | 1 |

**Table II** Haematological toxicity

| Dose (mg m$^{-2}$ day) | Number of patients | Number of evaluable courses | Leucocytes 1 2 3 4 | Neutrophils CTC grades 1 2 3 4 | Platelets 1 2 3 4 |
|---|---|---|---|---|---|
| 0.3 | 3 | 10 | 0 0 0 0 | 0 0 0 0 | 0 0 0 0 |
| 0.6 | 3 | 18 | 8 0 0 0 | 4 1 0 0 | 1 0 0 0 |
| 0.9 | 3 | 9 | 3 1 1 0 | 0 4 1 0 | 4 2 1 0 |
| 1.2 | 6 | 15 | 1 1 4 2 | 2 2 1 6 | 0 1 6 0 |
| 1.5 | 6 | 15 | 0 9 3 3 | 2 0 2 11 | 2 6 4 3 |

**Table III** Neutropenia and thrombocytopenia at the two highest dose levels

| | Median day of occurrence of grade 3–4 thrombocytopenia | Day nadir | Median number of days from grade 3–4 to recovery ⩾ grade 2 thrombocytopenia | Median day of occurrence of grade 3–4 neutropenia | Day nadir | Median number of days from grade 3–4 to recovery ⩾ grade 2 neutropenia |
|---|---|---|---|---|---|---|
| 1.2 mg m$^{-2}$ day$^{-1}$ | 15 (15–15)[a] | 15 (10–19)[a] | 7 (2–8)[a] | 15 (10–19)[b] | 22 (13–22)[b] | 10 (1–10)[b] |
| 1.5 mg m$^{-2}$ day$^{-1}$ | 15 (10–15)[b] | 15 (13–15)[b] | 8 (5–12)[b] | 10 (5–25)[c] | 15 (13–22)[c] | 11 (4–25)[c] |

[a]$n=6$. [b]$n=7$. [c]$n=13$.

**Figure 1** Comparison of the median ANC (**a**) and median platelet (**b**) counts of all given courses at doses of 1.2 (-●-) and 1.5 (+) mg m$^{-2}$ day$^{-1}$.

(range 0.01–1.7). The median number of days to recover from grade 3–4 neutropenia was 11 days (range 4–25). Three infectious complications were observed, two of them were life-threatening septic complications caused by gram-negative bacteria. In addition, thrombocytopenia grade 3–4 was seen in 7/15 courses (46%). At this dose of 1.5 mg m$^{-2}$ day$^{-1}$ the median platelet nadir was $62 \times 10^9$ l$^{-1}$ (range 10–116). The recovery of grade 3–4 thrombocytopenia occurred in 8 days (range 5–12). Thrombocytopenia was complicated by gastrointestinal bleeding in two patients. Anaemia occurred frequently but was never severe. Regularly, subsequent courses had to be postponed, owing to delayed recovery of myelosuppression. At the dose of 1.2 mg m$^{-2}$ day $^{-1}$ a treatment delay of 1 week was required in four courses and a delay of 2 weeks was required in two courses. At 1.5 mg m$^{-2}$ day $^{-1}$ a treatment delay of 1 week was necessary in three courses and a delay of 2 weeks in two courses.

Cumulative myelotoxicity was not observed. The pattern of myelosuppression during the first course predicted the pattern in all subsequent courses (Figure 2).

**Figure 2** Graph showing course of ANC (-□-) and platelets (+) during subsequent cycles with GI147211 of patient 16 at the dose of 1.5 mg m$^{-2}$ day$^{-1}$.

### *Non-haematological toxicity*

Overall, non-haematological toxicities were relatively mild. Nausea and vomiting were not dose-related and occurred in 30 (44%) and 15 (22%) of the given courses respectively and were never worse than grade 1–2. These symptoms were present only during the periods of drug administration and could easily be circumvented by the prophylactic use of standard anti-emetics. Alopecia was dose related, alopecia grade 1 was observed in three patients at the highest two dose levels. Mild headache was not dose-dependent and occurred in eight courses (12%). Prophylactic use of analgesics such as paracetamol during the days of drug administration prevented this symptom. No other toxicities were seen. There was no diarrhoea, mucositis, liver or renal toxicity.

### *Pharmacokinetics and kinetic–dynamic relationships*

Complete plasma sampling was obtained from all 18 patients on days 1 and 4 during the first course.

The pharmacokinetic data are summarised in Tables IV, V and VI. The AUC was linearly related to the dose (Figure 3). Total plasma clearance, determined on day 1, was 1014± 177.0 ml min$^{-1}$ m$^{-2}$ (mean±s.d.), $V_{dss}$ was 193± 45.9 l m$^{-2}$ (mean±s.d.), $t_{1/2}$ was 3.54±0.99 h (mean±s.d.) and mean residence time (MRT) was 3.53±0.93 h (mean±s.d.).

Significant sigmoid relationships were observed between the AUC on day 4 of treatment and the per cent decrease ANC (Figure 4). No significant influence of pretreatment on these relationships was observed (Figure 5).

The fraction of the drug excreted unchanged in the urine (fe) on day 1 was 0.14±0.07 and the renal clearance was 140±74 ml min$^{-1}$ m$^{-2}$ (Table IV).

*Responses* One partial response was seen in a patient with metastatic colorectal cancer. His tumour had previously been shown to be resistant to 5-FU/folinic acid. The remission achieved by GI147221 lasted 6 weeks. A minor response was observed after two courses in a patient with liver metastasis of a leiomyosarcoma of the stomach, previously progressive after two courses of doxorubicin/ifosfamide. The patient refused further treatment because the second course was complicated by a non-drug-related upper gastrointestinal bleeding. Stable disease was seen in ten patients.

### Discussion

The characterisation of the inhibition of topoisomerase I as the mechanism of action of CPT has resulted in the

**Table IV** Pharmacokinetic data after i.v. administration of GI147211 on day 1 of the first course

| *Patient number* | *Dose ($mg\ m^{-2}$)* | *Day* | *$AUC_{inf}$ ($ng\ ml^{-1}\ h$)* | *$C_{max}$ ($ng\ ml^{-1}$)* | *$t_{max}$ (h)* | *$t_{\frac{1}{2}}$ (h)* | *MRT (h)* | *Clearance ($ml\ min^{-1}\ m^{-2}$)* | *$V_{dss}$ ($l\ m^{-2}$)* |
|---|---|---|---|---|---|---|---|---|---|
| 1 | 0.3 | 1 | 4.93 | 3.63 | 0.42 | 2.96 | 2.89 | 1014 | 161 |
| 2 | 0.3 | 1 | 4.04 | 4.27 | 0.42 | 1.99 | 1.66 | 1238 | 104 |
| 3 | 0.3 | 1 | 4.77 | 3.73 | 0.42 | 2.63 | 3.33 | 1048 | 194 |
| | | Mean | 4.58 | 3.88 | 0.42 | 2.52 | 2.63 | 1100 | 153 |
| 4 | 0.6 | 1 | 10.10 | 9.16 | 0.42 | 3.35 | 4.01 | 990 | 223 |
| 5 | 0.6 | 1 | 15.63 | 9.41 | 0.42 | 2.81 | 3.74 | 640 | 134 |
| 6 | 0.6 | 1 | 14.00 | 11.46 | 0.42 | 2.80 | 3.52 | 714 | 140 |
| | | Mean | 13.25 | 10.01 | 0.42 | 2.98 | 3.76 | 781 | 166 |
| 7 | 1.2 | 1 | 17.57 | 17.33 | 0.42 | 3.62 | 3.01 | 1138 | 189 |
| 8 | 1.2 | 1 | 24.95 | 11.97 | 0.42 | 4.04 | 5.18 | 802 | 237 |
| 9 | 1.2 | 1 | 16.70 | 15.99 | 0.42 | 2.54 | 2.94 | 1198 | 193 |
| 10 | 1.2 | 1 | 15.05 | 12.83 | 0.42 | 2.40 | 3.04 | 1329 | 223 |
| 11 | 1.2 | 1 | 19.86 | 14.11 | 0.25 | 5.10 | 3.98 | 1007 | 225 |
| 12 | 1.2 | 1 | 19.42 | 17.65 | 0.42 | 2.97 | 2.66 | 1030 | 149 |
| | | Mean | 18.93 | 14.98 | 0.39 | 3.45 | 3.47 | 1084 | 203 |
| 13 | 1.5 | 1 | 24.66 | 15.08 | 0.25 | 4.98 | 5.11 | 1014 | 296 |
| 14 | 1.5 | 1 | – | 27.48 | 0.42 | – | – | – | – |
| 15 | 1.5 | 1 | 24.39 | 19.21 | 0.42 | 4.62 | 3.57 | 1025 | 204 |
| 16 | 1.5 | 1 | 23.32 | 19.53 | 0.25 | 4.37 | 3.32 | 1072 | 197 |
| 17 | 1.5 | 1 | 22.64 | 18.91 | 0.25 | 4.61 | 3.65 | 1104 | 225 |
| 18 | 1.5 | 1 | 28.37 | 15.90 | 0.75 | 4.42 | 3.78 | 881 | 186 |
| | | Mean | 24.68 | 19.35 | 0.39 | 4.60 | 3.89 | 1019 | 222 |

$AUC_{inf}$, AUC after extrapolation to infinity; $C_{max}$, maximal plasma concentration; $t_{max}$, time to maximal plasma concentration; $t_{\frac{1}{2}}$, elimination half-life; clearance, total plasma clearance; $V_{dss}$, apparent volume of distribution at steady state; MRT, mean residence time.

**Table V** Pharmacokinetic data after i.v. administration of GI147211 on day 4 of the first course

| *Patient number* | *Dose ($mg\ m^{-2}$)* | *Day* | *$AUC_{inf}$ ($ng\ ml^{-1}\ h$)* | *$C_{max}$ ($ng\ ml^{-1}$)* | *$t_{max}$ (h)* | *$t_{\frac{1}{2}}$ (h)* | *MRT (h)* | *Clearance ($ml\ min^{-1}\ m^{-2}$)* | *$V_{dss}$ ($l\ m^{-2}$)* |
|---|---|---|---|---|---|---|---|---|---|
| 1 | 0.3 | 4 | 6.19 | 4.73 | 0.25 | 4.82 | 4.62 | 808 | 207 |
| 2 | 0.3 | 4 | 6.79 | 4.30 | 0.25 | 2.34 | 4.73 | 736 | 199 |
| 3 | 0.3 | 4 | 7.46 | 5.11 | 0.42 | 3.81 | 5.30 | 670 | 199 |
| | | Mean | 6.81 | 4.71 | 0.31 | 3.65 | 4.88 | 738 | 202 |
| 4 | 0.6 | 4 | 11.21 | 7.60 | 0.42 | 3.42 | 4.89 | 892 | 246 |
| 5 | 0.6 | 4 | 15.10 | 8.13 | 0.42 | 3.78 | 5.38 | 662 | 203 |
| 6 | 0.6 | 4 | 15.94 | 9.29 | 0.25 | 4.15 | 6.35 | 6.27 | 224 |
| | | Mean | 14.08 | 8.34 | 0.36 | 3.78 | 5.54 | 727 | 224 |
| 7 | 1.2 | 4 | 20.59 | 18.66 | 0.25 | 3.02 | 3.06 | 971 | 163 |
| 8 | 1.2 | 4 | 37.83 | 20.03 | 0.42 | 4.07 | 5.62 | 529 | 167 |
| 9 | 1.2 | 4 | 21.10 | 16.21 | 0.42 | 2.70 | 3.47 | 948 | 182 |
| 10 | 1.2 | 4 | 14.84 | 9.62 | 0.42 | 2.20 | 3.96 | 1348 | 299 |
| 11 | 1.2 | 4 | 26.62 | 12.74 | 0.42 | 6.99 | 7.06 | 802 | 307 |
| 12 | 1.2 | 4 | 16.96 | 11.63 | 0.42 | 4.99 | 4.44 | 1179 | 291 |
| | | Mean | 22.99 | 14.82 | 0.39 | 3.99 | 4.60 | 963 | 235 |
| 13 | 1.5 | 4 | 26.92 | 20.02 | 0.42 | 4.95 | 5.06 | 929 | 261 |
| 14 | 1.5 | 4 | 29.51 | 23.81 | 0.42 | 3.44 | 2.56 | 847 | 117 |
| 15 | 1.5 | 4 | 30.85 | 25.41 | 0.25 | 4.35 | 3.04 | 810 | 144 |
| 16 | 1.5 | 4 | 34.51 | 32.19 | 0.42 | 5.62 | 4.47 | 724 | 178 |
| 17 | 1.5 | 4 | 24.85 | 21.18 | 0.25 | 5.82 | 5.75 | 1006 | 319 |
| 18 | 1.5 | 4 | 52.70 | 28.25 | 0.42 | 4.43 | 4.74 | 474 | 126 |
| | | Mean | 33.22 | 25.14 | 0.36 | 4.77 | 4.27 | 798 | 191 |

$AUC_{inf}$, AUC after extrapolation to infinity; $C_{max}$, maximal plasma concentration; $t_{max}$, time to maximal plasma concentration; $t_{\frac{1}{2}}$, elimination half-life; clearance, total plasma clearance; $V_{dss}$, apparent volume of distribution at steady state; MRT, mean residence time.

**Table VI** Pharmacokinetic data after i.v. administration of GI147211 on days 1 and 4 of the first course

| *Patient number* | *Day* | *fe* | *Clr (ml min$^{-1}$)* | *Clr* | *Day* | *fe* | *Clr (ml min$^{-1}$)* | *Clr (ml min$^{-1}$ m$^{-2}$)* |
|---|---|---|---|---|---|---|---|---|
| 1 | 1 | 0.114 | 232 | 116 | 4 | 0.109 | 175 | 88 |
| 2 | 1 | 0.081 | 184 | 101 | 4 | 0.095 | 128 | 70 |
| 3 | 1 | 0.212 | 333 | 222 | 4 | 0.428 | 430 | 287 |
| 4 | 1 | 0.115 | 231 | 115 | 4 | – | – | – |
| 5 | 1 | 0.156 | 161 | 101 | 4 | 0.181 | 192 | 120 |
| 6 | 1 | 0.149 | 194 | 107 | 4 | 0.197 | 225 | 123 |
| 7 | 1 | 0.106 | 201 | 122 | 4 | 0.122 | 196 | 119 |
| 8 | 1 | 0.101 | 151 | 84 | 4 | 0.219 | 212 | 118 |
| 9 | 1 | 0.088 | 203 | 107 | 4 | 0.133 | 241 | 127 |
| 10 | 1 | 0.085 | 226 | 113 | 4 | 0.128 | 344 | 172 |
| 11 | 1 | 0.116 | 208 | 119 | 4 | 0.239 | 314 | 179 |
| 12 | 1 | 0.347 | 663 | 358 | 4 | 0.210 | 459 | 248 |
| 13 | 1 | – | – | – | 4 | – | – | – |
| 14 | 1 | 0.191 | – | – | 4 | – | – | – |
| 15 | 1 | 0.135 | 280 | 150 | 4 | – | – | – |
| 16 | 1 | – | – | – | 4 | 0.177 | 218 | 128 |
| 17 | 1 | – | – | – | 4 | 0.090 | 182 | 91 |
| 18 | 1 | – | – | – | 4 | 0.125 | 101 | 59 |
| | Mean | 0.143 | 251 | 140 | | 0.0175 | 244 | 138 |
| | s.d. | 0.070 | 133 | 74 | | 0.087 | 106 | 64 |
| | CV (%) | 49 | 53 | 53 | | 50 | 43 | 47 |

fe, fraction of the drug excreted unchanged; Clr, renal clearance.

development of several semisynthetic CPT analogues, of which some are under extensive clinical investigation. This is the first report of a clinical phase I study with GI147211.

The dose-limiting toxicity of GI147211 administered as a 30 min i.v infusion for 5 consecutive days in patients with solid tumours was neutropenia in conjunction with thrombocytopenia. The dose of 1.2 mg m$^{-2}$ day$^{-1}$ was considered the maximally tolerated dose. At this dose the onset of neutropenia grade 3–4 occurred between days 10 and 19, with a median ANC nadir of $1.29 \times 10^9$ l$^{-1}$ (range 0.08–5.6). The median day of the platelet nadir and the first day of grade 3–4 toxicity was day 15. The median number of days to recovery was 7 (range 2–8). The recovery of neutropenia was even more prolonged, it lasted 10 days (range 1–10). Owing to this prolonged myelosuppression subsequent courses had to be postponed in 6/15 courses at this dose level of 1.2 mg m$^{-2}$ day$^{-1}$. At the dose of 1.5 mg m$^{-2}$ day$^{-1}$ the dose-limiting toxicity was reached. At this dose grade 3–4 neutropenia already was noticed as early as day 10 (range 5–25) and lasted for 11 days (4–25) with a median ANC nadir of $0.09 \times 10^9$ l$^{-1}$ (range 0.01–1.7). This deep and prolonged recovery from neutropenia was complicated by three septic episodes, and resulted in treatment delay in 5 of the 15 courses given at this dose level. Although the depth, the time of occurrence and recovery from thrombocytopenia at this dose level was equal to the dose of 1.2 mg m$^{-2}$ day$^{-1}$, at this dose the thrombocytopenia was complicated in two courses by gastrointestinal bleeding.

As only one patient was heavily pretreated (ten courses of anthracyclin-containing chemotherapy) at the higher dose levels, these treatment delays were not related to prior myelosuppressive therapies. There were no indications of cumulative myelosuppression, the pattern of myelosuppression in subsequent courses was equal (see Figure 2). The use of haematopoietic growth factors might be helpful in preventing infections, but will be of limited value in further dose escalation of GI147211, since dose-limiting thrombocytopenia occurred in conjunction with neutropenia.

The pharmacokinetic analysis reveals moderate interpatient variability. The pharmacokinetic data, obtained on days 1 and 4 of course one, demonstrate limited intrapatient variability. There was a linear relationship between the AUC

**Figure 3** Relationship between the dose m$^{-2}$ and the area under the curve (AUC) of GI147211 determined on day 4 of course 1.

**Figure 4** Relationship between the AUC of GI147211 determined on day 4 of the first course and the per cent decrease in AUC. The sigmoid $E_{max}$ model was applied.

**Figure 5** Relationship between the AUC of GI147211 determined on day 4 of the first course and the per cent decrease in AUC, in minimally and heavily pretreated patients. $EC_{50}$ value in minimally pretreated patients, 18.95 (-■-). $EC_{50}$ value in heavily pretreated patients, 18.00 (-▲-).

and the administered dose. A steep sigmoid relationship was observed between the AUC on day 4 and per cent decrease ANC, indicating that the AUC is predictive for the myelosuppression.

In this study one short-lasting partial response was noted in a patient with metastatic colorectal cancer. This fits with the observations of activity of GI147211 in preclinical models against colorectal cancer. In addition, it is of interest that we observed a minor response in a patient with leiomyosarcoma. In a recently reported phase II study in metastatic soft tissue sarcoma the topoisomerase I inhibitor topotecan only showed responses in patients with leiomyosarcoma (Eisenhauer *et al.*, 1994).

In phase I studies with a daily × 5 schedule of topotecan the dose-limiting toxicity was also myelosuppression, predominantly severe neutropenia of brief duration not necessitating treatment delays (Rowinsky *et al.*, 1992; Verweij *et al.*, 1993). Thrombocytopenia mainly occurred in prolonged continuous regimens and the myelosuppression was not cumulative (Hochster *et al.*, 1994). As the daily × 5 schedule appeared to be most active in early phase I studies many different phase II studies were initiated with this scheme. Although a randomised comparison is obviously lacking, GI147211 seems to induce more prolonged myelosuppression than topotecan. Presumably related to this, in contrast to topotecan, GI147211 administration necessitated relatively frequent delays of retreatment. These human data therefore confirm preclinical studies in bone marrow cultures where GI147211 was found to be more myelotoxic than topotecan. In preclinical studies this increased myelotoxicity of GI147211 seems to coincide with more anti-tumour activity (Emerson *et al.*, 1995).

The other topoisomerase I inhibitor in a well-advanced stage of clinical development, Irinotecan (CPT-11) induces neutropenia in addition to diarrhoea. Diarrhoea was not observed at all for GI147211. Unlike GI147211, which is the active compound, CPT-11 is a prodrug. CPT-11 has to be converted to the active metabolite SN-38. It has been hypothesised that the conversion in the intestinal mucosa might be responsible for the diarrhoea. The fact that such a conversation is not required for GI147211 may result in relatively low intestinal mucosal drug levels as compared with SN-38, and thereby less mucosal damage.

Preclinical data have indicated that topoisomerase I inhibitors, like topoisomerase II inhibitors, demonstrate more efficacy with prolonged continuous exposure (Burris *et al.*, 1992; Giovanella *et al.*, 1989; Houghton *et al.*, 1993). Therefore, future development of GI147211 will be focused on prolonged infusions, and the apparent bioavailability of oral administration will be determined.

The recommended dose for phase II studies with a daily × 5 intravenous schedule is 1.2 mg $m^{-2}$ $day^{-1}$ repeated every 3 weeks. Phase II studies in various tumour types have recently been initiated.

### References

BURRIS HA, HANAUSKE AR, JOHNSON RK, MASHALL MH, KUHN JG, HILSENBEEK SG AND VON HOFF DD. (1992). Activity of topotecan a new topoisomerase I inhibitor, against human colony-forming units in vitro. *J. Natl Cancer Inst.*, **84,** 1816–1820.

CREAVEN PJ, ALLEN LM AND MUGGIA FM. (1972). Plasma camptothecin (NSC 100880) levels during a 5-days course of treatment: relation to dose and toxicity. *Cancer Chem. Rep.*, **56,** 573–578.

CREEMERS GJ, LUND B AND VERWEIJ J. (1994). Topoisomerase I inhibitors: Topotecan and irinotecan. *Cancer Treat. Rev.*, **20,** 73–96.

COVEY JM, JAXEL C AND KOHN KW. (1989). Protein-link DNA strand break induced in mammalian cells by camptothecin, an inhibitor of topoisomerase I. *Cancer Res.*, **49,** 5016–5022.

D'ARPA P AND LIU LF. (1989). Topoisomerase-targeting antitumor drugs. *Biochim. Biophys. Acta*, **989,** 163–177.

EISENHAUER EA, WAINMAN N, BOOS G, MACDONALD D, BRAMWELL V AND NCI CANADA CLINICAL TRIALS GROUP. (1994). Phase II trials of topotecan in patients(pts) with malignant glioma and soft tissue sarcoma. *Proc. Am. Assoc. Clin. Onc.*, **13,** 488.

EMERSON DL, VUONG A, MCINTYRE MS, CROOM DK AND BESTERMAN JM. (1993). *In vivo* efficacy of two new water-soluble camptothecin analogs in the human cancer xenograft model. *Proc. Am. Assoc. Cancer Res.*, **34,** 419.

EMERSON DL, MCINTYRE G, LUZZIO MJ AND WISSEL PS. (1994). Preclinical antitumor activity of a novel water-soluble camptothecin analog (GI147211C). *Ann. Oncol.*, **5,** (suppl. 5), 185.

EMERSON DL, BESTERMAN JM, BROWN HR, EVANS MG, LEITNER PP, LUZZIO MJ, SCHAFFER JE, STERNBACH DD, UEHLING D AND VUONG A. (1995). In vivo antitumor activity of two new seven-substituted water-soluble camptothecin analogues. *Cancer Res.*, **55,** 603–609.

ENG WK, FAUCETTE L, JOHNSON RK AND STERNGHANZ R. (1988). Evidence that topoisomerase I is necessary for the cytotoxic effects of camptothecin. *Mol. Pharmacol.*, **34,** 755–760.

GIOVANELLA BC, STEHLIN JS, WALL ME, WANI MC, NICHOLAS AW, LIU FL, SILBER R AND POTMESIL M. (1989). DNA topoisomerase I-targeted chemotherapy of human colon cancer in xenografts. *Science*, **246,** 1046–1048.

GOTTLIEB JA, GUARINO AM, CALL JB, OLIVERIO VT AND BLOCK JB. (1970). Preliminary pharmacologic and clinical evaluation of camptothecin sodium (NSC 100880). *Cancer Chem. Rep.*, **54,** 461–470.

HIRABAYASHI N, KIM R, NISHIYAMA M, AOGI K, SAEKI S, TOGE T AND OKADA K. (1992). Tissue expression of topoisomerase I and II in digestive tract cancers and adjacent normal tissues. *Proc. Am. Assoc. Cancer Res.*, **33,** 2603.

HOCHSTER H, LIEBES L, SPEYER J, SORICH J, TAUBES B, ORATZ R, WERN EJ, CHACHOUA A, RAPHAEL B, VINCI RE AND BLIM RH. (1994). Phase I trial of low-dose continuous topotecan infusion in patients with cancer: an active and well-tolerated regimen. *J. Clin. Oncol.*, **12,** 553–559.

HORWITZ SP AND HORWITZ HS. (1973). Effects of camptothecin on the breakage and repair of DNA during the cell cycle. *Cancer Res.*, **33,** 2834–2836.

HOUGHTON PJ, CHESHIRE PJ, HALLMAN JC, BISSERY MC, MATHIEU-BOUÉ A AND HOUGHTON J. (1993). Therapeutic efficacy of the topoisomerase I inhibitor 7-ethyl-10-(4[1-piperidino]-1-piperidino)-carbonyloxycamptothecin against human tumor xenografts: lack of cross-resistance in vivo tumors with acquired resistance to the topoisomerase I inhibitor 9-dimethylamino-methyl-10-camptothecin. *Cancer Res.*, **53**, 2823–2829.

HSIANG YH AND LIU LF. (1988). Identification of mammalian DNA topoisomerase I as an intracellular target of the anticancer drug camptothecin. *Cancer Res.*, **48**, 1722–1726.

HSIANG YH, HERTZBERG R, HECHT S AND LIU LF. (1985). Camptothecin induces protein-linked DNA breaks via mammalian DNA topoisomerase I. *J. Biol. Chem.*, **260**, 14873–14878.

MUGGIA FM, CREAVEN PJ, HANSEN HH, COHEN MH AND SELANRING OS. (1972). Phase I clinical trial of weekly and daily treatment with camptothecin (NSC 100880): correlation with preclinical studies. *Cancer Chem. Rep.*, **56**, 515–521.

NATIONAL CANCER INSTITUTE. (1988). *Guidelines for Reporting of Adverse Drug Reactions*. Division of Cancer Treatment, National Cancer Institute: Bethesda, MD.

POTMESIL M. (1994). Camptothecins: from bench research to hospital wards. *Cancer Res.*, **54**, 1431–1439.

POTMESIL M, HSIANG YH, LIU LF, BANK B, GROSSBERG H, KIRSCHENBAUM S, FORLENZAR TJ, RENZINCH A, KANGANIS D, KNOWLES D, TRAGANOS F AND SILBER R. (1988). Resistance of human leukemia and normal lymphocytes to drug induced DNA cleavage and low levels of topoisomerase II. *Cancer Res.*, **48**, 3537–3543.

ROWINSKY EK, GROCHOW LB, HENDRICKS CB, ETTINGER DS, FORASTIERE AA, HUROWITZ LA, MCGUIRE WP, SATORIUS SE, LUBEJKO BG, KAUFMANN SH AND DONEHOWER RC. (1992). Phase I and pharmacologic study of topotecan: a novel topoisomerase I inhibitor. *J. Clin. Oncol.*, **10**, 647–656.

SLICHENMYER WJ, ROWINSKY EK, DONEHOWER RCAND KAUFMANN SH. (1993). The current status of camptothecin analogues as antitumor agents. *J. Natl Cancer Inst.*, **85**, 271–291.

STAFFORD CG AND ST. CLAIRE III RL. (1995). High-performance liquid chromatographic analysis of the lactone and carboxylate forms of a topoisomerase I inhibitor (the antitumor drug GI147211) in plasma. *J. Chromatogr.*, **663**, 119–126.

VERWEIJ J, LUND B, BEIJNEN J, PLANTING A, DE BOER-DENNERT M, KOIER I, ROSING H AND HANSSEN H. (1993). Phase I and pharmacokinetics study of topotecan, a new topoisomerase I inhibitor. *Ann. Oncol.*, **4**, 673–678.

WORLD HEALTH ORGANIZATION. (1979). *WHO Handbook for Reporting Results of Cancer Treatment*. WHO offset publication No. 40. World Health Organization: Geneva, Switzerland.


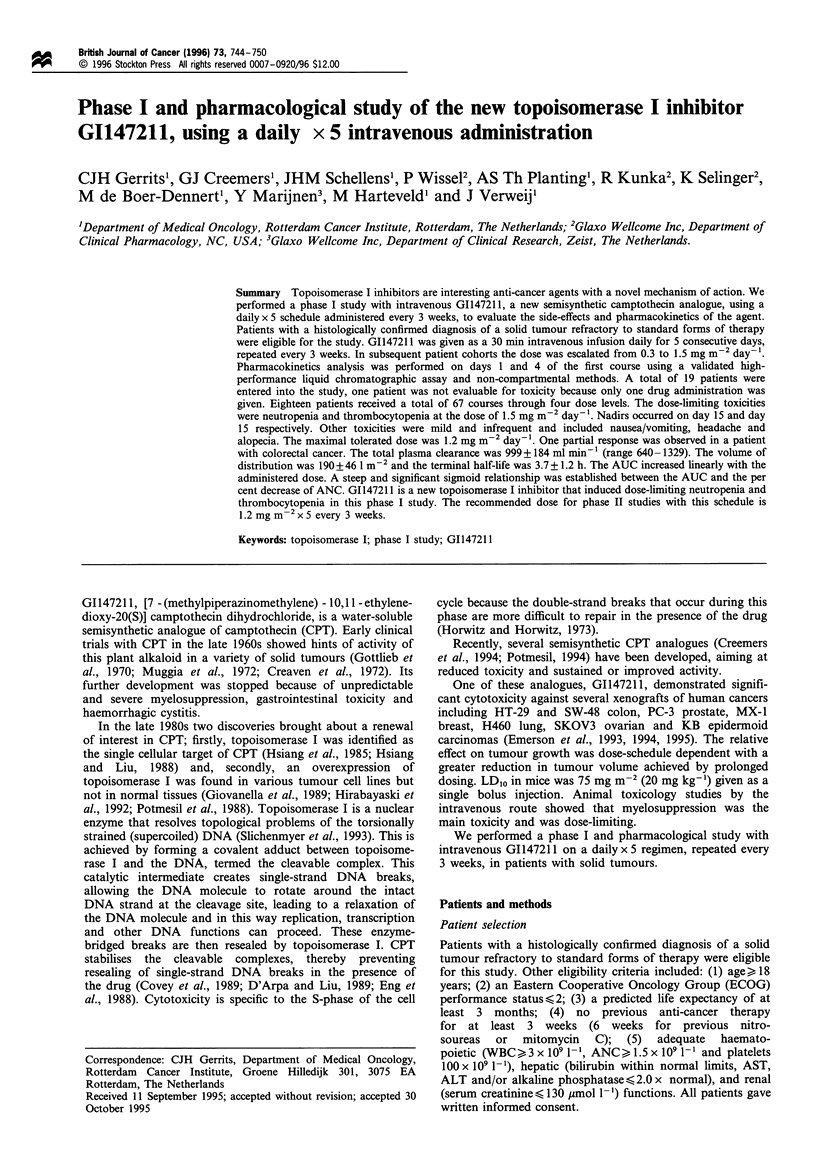

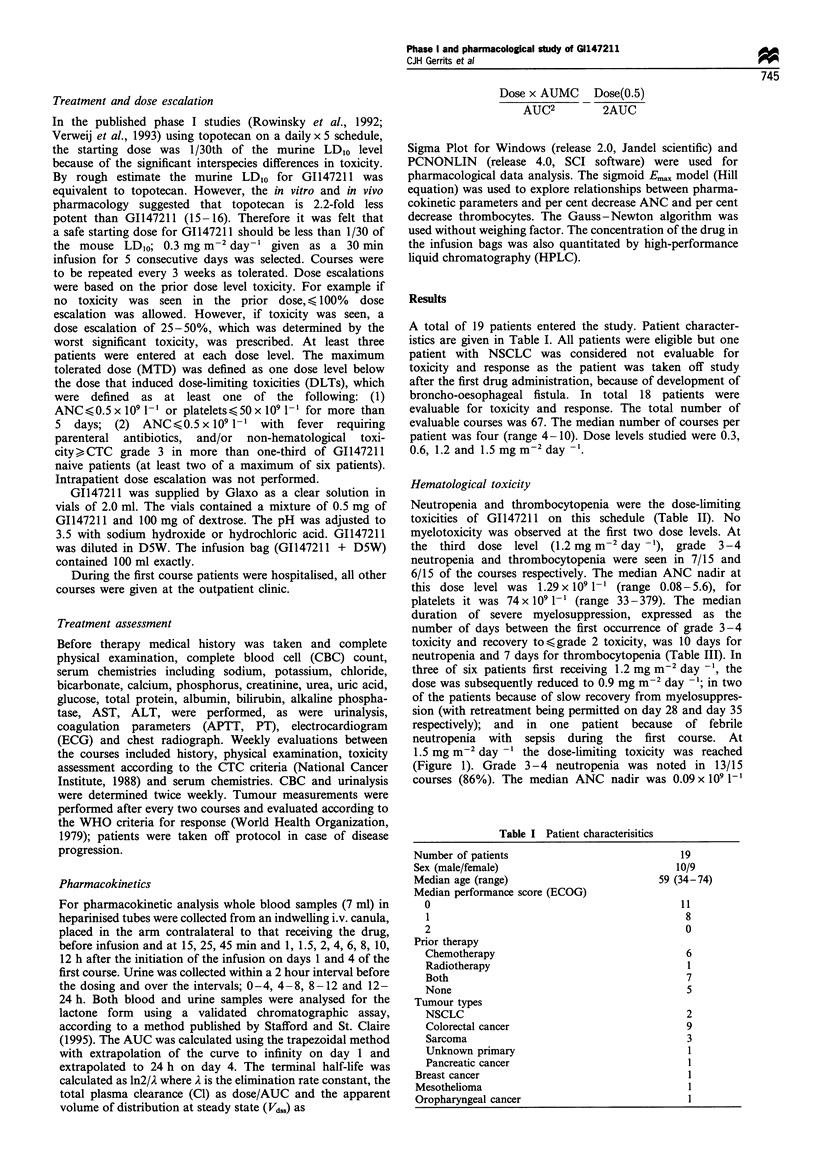

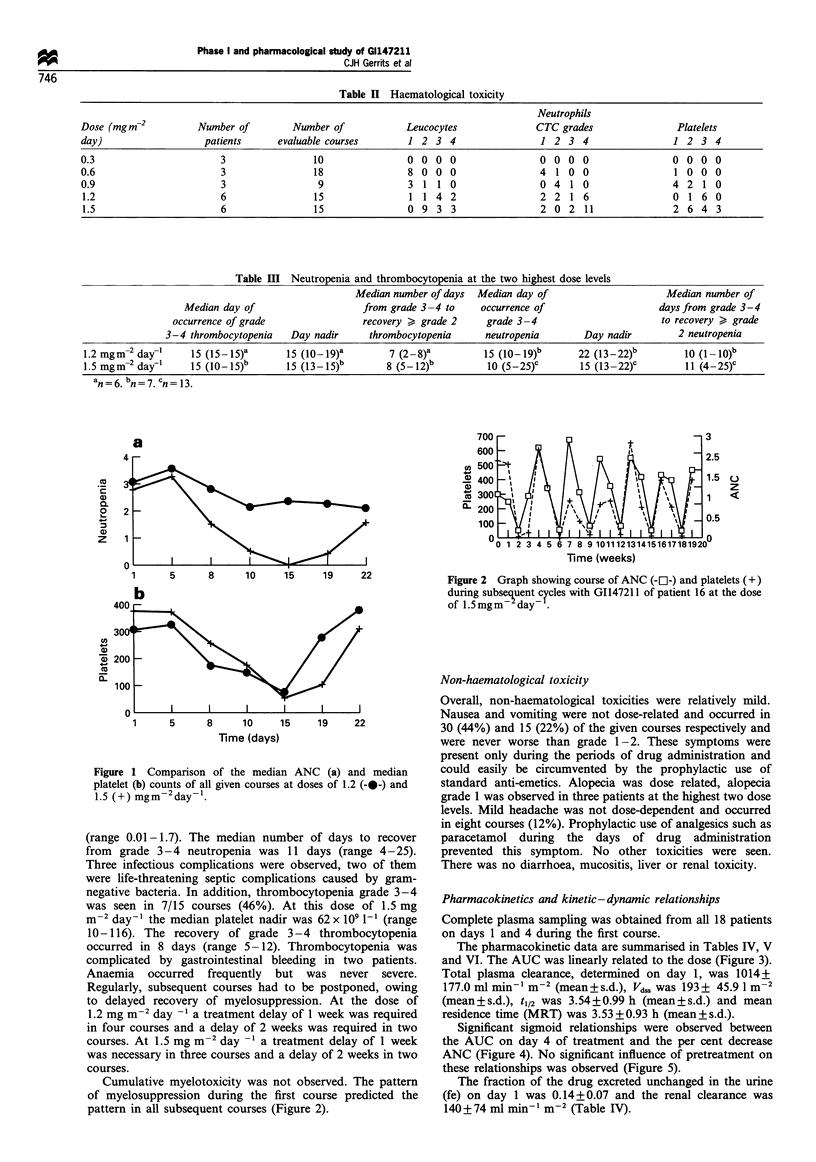

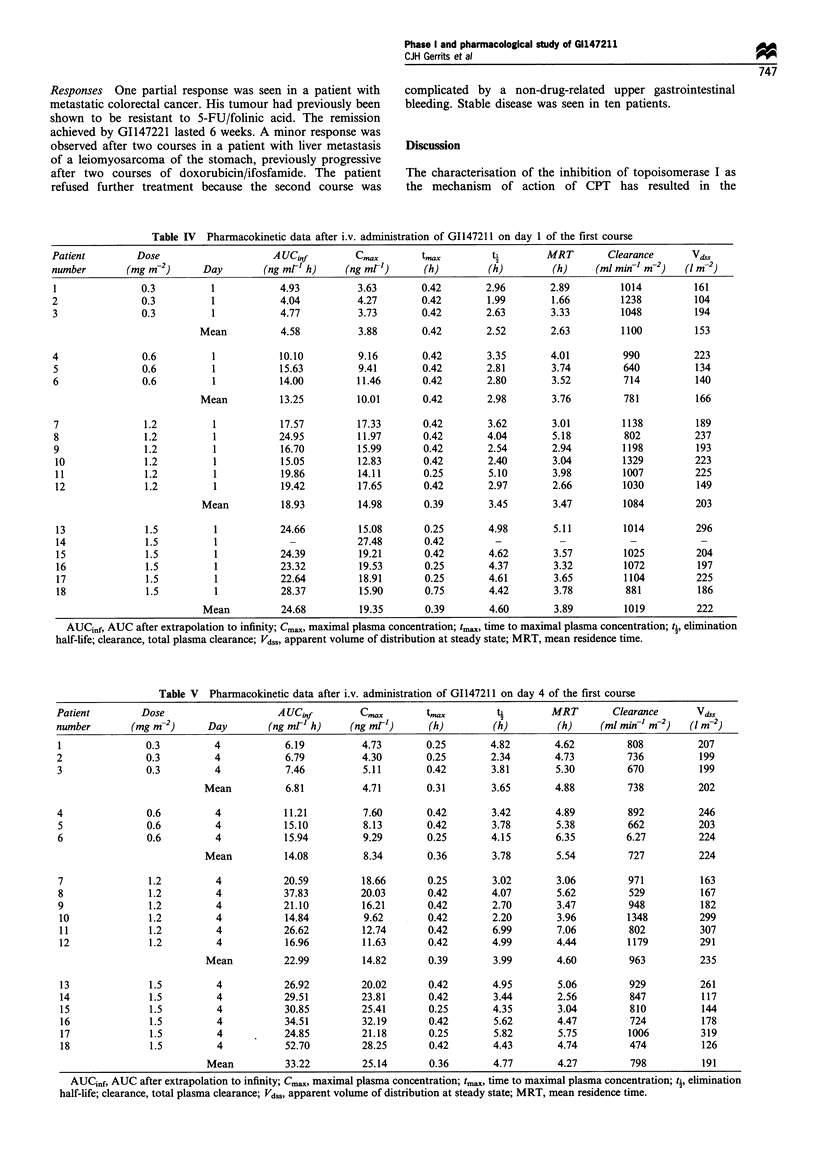

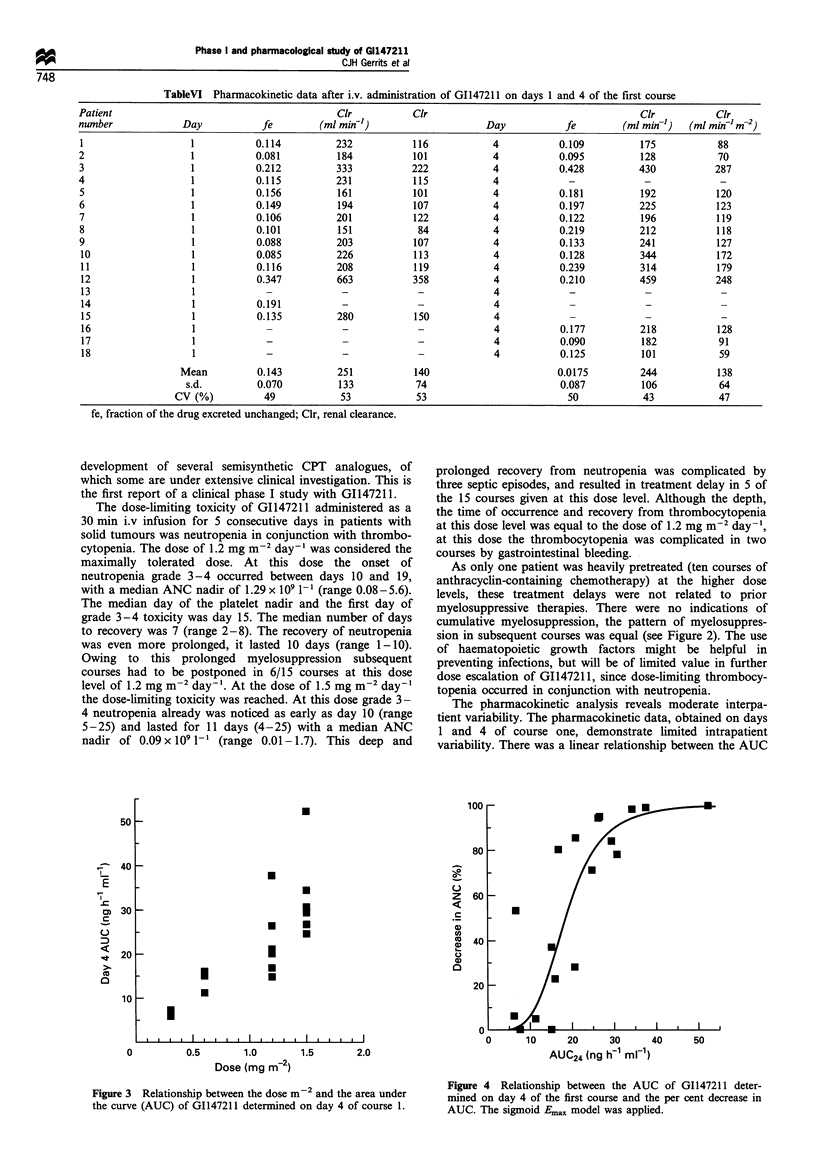

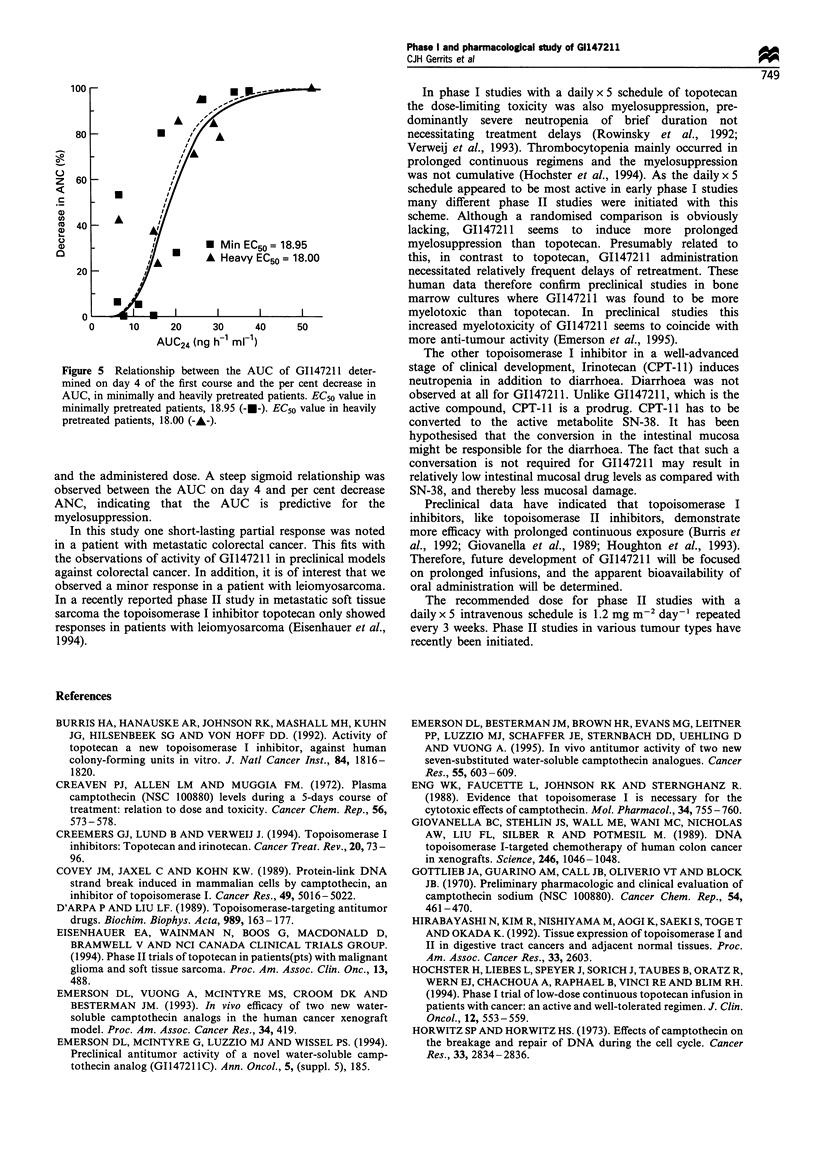

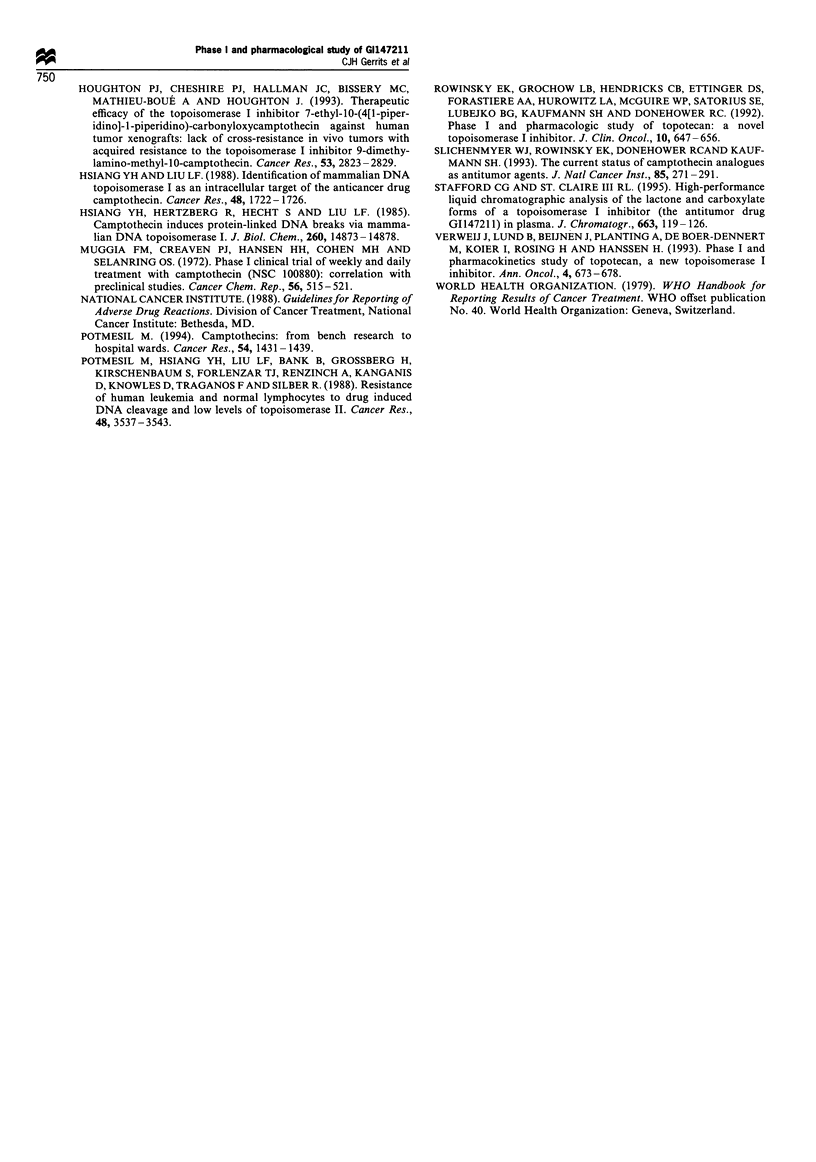


## References

[OCR_00855] Burris H. A., Hanauske A. R., Johnson R. K., Marshall M. H., Kuhn J. G., Hilsenbeck S. G., Von Hoff D. D. (1992). Activity of topotecan, a new topoisomerase I inhibitor, against human tumor colony-forming units in vitro.. J Natl Cancer Inst.

[OCR_00871] Covey J. M., Jaxel C., Kohn K. W., Pommier Y. (1989). Protein-linked DNA strand breaks induced in mammalian cells by camptothecin, an inhibitor of topoisomerase I.. Cancer Res.

[OCR_00860] Creaven P. J., Allen L. M., Muggia F. M. (1972). Plasma camptothecin (NSC-100880) levels during a 5-day course of treatment: relation to dose and toxicity.. Cancer Chemother Rep.

[OCR_00866] Creemers G. J., Lund B., Verweij J. (1994). Topoisomerase I inhibitors: topotecan and irenotecan.. Cancer Treat Rev.

[OCR_00878] D'Arpa P., Liu L. F. (1989). Topoisomerase-targeting antitumor drugs.. Biochim Biophys Acta.

[OCR_00901] Emerson D. L., Besterman J. M., Brown H. R., Evans M. G., Leitner P. P., Luzzio M. J., Shaffer J. E., Sternbach D. D., Uehling D., Vuong A. (1995). In vivo antitumor activity of two new seven-substituted water-soluble camptothecin analogues.. Cancer Res.

[OCR_00905] Eng W. K., Faucette L., Johnson R. K., Sternglanz R. (1988). Evidence that DNA topoisomerase I is necessary for the cytotoxic effects of camptothecin.. Mol Pharmacol.

[OCR_00911] Giovanella B. C., Stehlin J. S., Wall M. E., Wani M. C., Nicholas A. W., Liu L. F., Silber R., Potmesil M. (1989). DNA topoisomerase I--targeted chemotherapy of human colon cancer in xenografts.. Science.

[OCR_00917] Gottlieb J. A., Guarino A. M., Call J. B., Oliverio V. T., Block J. B. (1970). Preliminary pharmacologic and clinical evaluation of camptothecin sodium (NSC-100880).. Cancer Chemother Rep.

[OCR_00927] Hochster H., Liebes L., Speyer J., Sorich J., Taubes B., Oratz R., Wernz J., Chachoua A., Raphael B., Vinci R. Z. (1994). Phase I trial of low-dose continuous topotecan infusion in patients with cancer: an active and well-tolerated regimen.. J Clin Oncol.

[OCR_00936] Horwitz S. B., Horwitz M. S. (1973). Effects of camptothecin on the breakage and repair of DNA during the cell cycle.. Cancer Res.

[OCR_00943] Houghton P. J., Cheshire P. J., Hallman J. C., Bissery M. C., Mathieu-Boué A., Houghton J. A. (1993). Therapeutic efficacy of the topoisomerase I inhibitor 7-ethyl-10-(4-[1-piperidino]-1-piperidino)-carbonyloxy-camptothecin against human tumor xenografts: lack of cross-resistance in vivo in tumors with acquired resistance to the topoisomerase I inhibitor 9-dimethylaminomethyl-10-hydroxycamptothecin.. Cancer Res.

[OCR_00957] Hsiang Y. H., Hertzberg R., Hecht S., Liu L. F. (1985). Camptothecin induces protein-linked DNA breaks via mammalian DNA topoisomerase I.. J Biol Chem.

[OCR_00952] Hsiang Y. H., Liu L. F. (1988). Identification of mammalian DNA topoisomerase I as an intracellular target of the anticancer drug camptothecin.. Cancer Res.

[OCR_00965] Muggia F. M., Creaven P. J., Hansen H. H., Cohen M. H., Selawry O. S. (1972). Phase I clinical trial of weekly and daily treatment with camptothecin (NSC-100880): correlation with preclinical studies.. Cancer Chemother Rep.

[OCR_00975] Potmesil M. (1994). Camptothecins: from bench research to hospital wards.. Cancer Res.

[OCR_00980] Potmesil M., Hsiang Y. H., Liu L. F., Bank B., Grossberg H., Kirschenbaum S., Forlenza T. J., Penziner A., Kanganis D., Forlenzar T. J. (1988). Resistance of human leukemic and normal lymphocytes to drug-induced DNA cleavage and low levels of DNA topoisomerase II.. Cancer Res.

[OCR_00987] Rowinsky E. K., Grochow L. B., Hendricks C. B., Ettinger D. S., Forastiere A. A., Hurowitz L. A., McGuire W. P., Sartorius S. E., Lubejko B. G., Kaufmann S. H. (1992). Phase I and pharmacologic study of topotecan: a novel topoisomerase I inhibitor.. J Clin Oncol.

[OCR_00995] Slichenmyer W. J., Rowinsky E. K., Donehower R. C., Kaufmann S. H. (1993). The current status of camptothecin analogues as antitumor agents.. J Natl Cancer Inst.

[OCR_00999] Stafford C. G., St Claire R. L. (1995). High-performance liquid chromatographic analysis of the lactone and carboxylate forms of a topoisomerase I inhibitor (the antitumor drug GI147211) in plasma.. J Chromatogr B Biomed Appl.

[OCR_01003] Verweij J., Lund B., Beijnen J., Planting A., de Boer-Dennert M., Koier I., Rosing H., Hansen H. (1993). Phase I and pharmacokinetics study of topotecan, a new topoisomerase I inhibitor.. Ann Oncol.

